# Diabetes Induces Accumulation of Carbonylated Proteins in the Rat Retinal Pigment Epithelium Independently of Oxidative Stress

**DOI:** 10.1096/fba.2025-00230

**Published:** 2026-01-21

**Authors:** Elena Morales‐Ramírez, Juan David Villeda‐González, Gustavo Sánchez‐Chávez, Rocío Salceda

**Affiliations:** ^1^ Departamento de Neurodesarrollo y Fisiología, Instituto de Fisiología Celular Universidad Nacional Autónoma de México Ciudad de México México

**Keywords:** antioxidant, carbonylation, glutathione, hyperglycemia, Nrf2, oxidative stress, retinal pigment epithelium

## Abstract

Diabetic retinopathy (DR) is a major complication of diabetes mellitus. Growing evidence shows that hyperglycemia causes not only microvascular damage but also retinal neural dysfunction. Although different metabolic pathways have been implicated, the exact mechanism behind retinal degeneration remains unclear. Hyperglycemic stimuli have been shown to reduce the function of the retinal blood–barrier (BRB) in both diabetic humans and animals. As part of the BRB, the retinal pigment epithelium (RPE) plays a key role in retinal function by regulating the flow of metabolites and ions between the choroidal blood supply and the outer retina, and by supporting photoreceptor cell functions. Therefore, RPE dysfunction can lead to retinal injury. To understand the role of RPE in DR, we studied oxidative stress in the RPE at the early onset of streptozotocin‐induced diabetes in rats. We found a 60% increase in lipoperoxidation at 45 days of diabetes, along with a 50% reduction in ascorbic acid content. Oxidized proteins were significantly increased after 20 and 45 days of diabetes induction, and changes in cell–cell contacts were observed. Despite these findings, superoxide dismutase activity was greatly increased at 45 days of diabetes, while Nrf2 expression and levels of total and reduced glutathione, key regulators of cellular antioxidant capacity, were similar in control and diabetic rat RPE. Moreover, the increase in oxidized proteins was not affected by the antioxidant quercetin nor by the NOS inhibitor L‐NAME. These findings suggest that protein carbonylation may impair protein function or turnover, which in turn leads to RPE damage.

## Graphical abstract

The retinal pigment epithelium (RPE) plays a crucial role in retinal function, regulating the flow of metabolites and ions between the choroidal blood supply and the outer retina. Early hyperglycemia induced by streptozotocin results in elevated levels of oxidized proteins and lipid peroxidation. Despite this, the RPE possesses a high capacity to maintain its redox status, keeping Nrf2 expression and total and reduced glutathione levels unchanged. The increase in oxidized proteins may be due to alterations in degradation mechanisms or to an unfolded protein response.

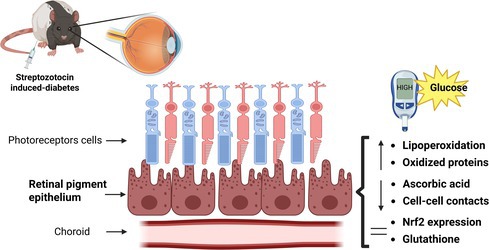

## 1 Introduction

Diabetes mellitus is a chronic disease marked by the absence of insulin or a decrease in its action, leading to high blood glucose levels (hyperglycemia). Diabetic retinopathy (DR) is the most common complication of diabetes [1], characterized by a progressive neuronal dysfunction and disruption of the blood‐retinal barrier [2, 3], caused by persistent hyperglycemia [4, 5].

Although various metabolic pathways, including oxidative/nitrosative stress, have been shown to play a role in the development of DR [6, 7, 8, 9, 10], its exact cause remains unknown.

In addition to the retinal vessels, blood glucose enters the retina through the retinal pigment epithelium (RPE), which forms the posterior part of the blood–retinal barrier (BRB) and plays a crucial role in supporting retinal function [11, 12]. The RPE is a single layer of hexagonal epithelial cells located between the outer segments of photoreceptor cells and the choroidal vasculature. In addition to being part of the BRB through tight junctions at the apical periphery of its basolateral side, the RPE is essential for maintaining the structure and function of the neural retina by protecting against photooxidation, converting all‐trans‐retinal into 11‐cis‐retinal, phagocytosing shed photoreceptor membranes, and secreting various important factors that support the structural integrity of the retina [13, 14, 15, 16].

Because of its high metabolic activity and constant light exposure, the RPE is at high risk of oxidative/nitrosative injury. Furthermore, we previously found a significant increase in nitric oxide (NO) content in diabetic RPE [17]. Given the importance of the RPE to retinal health and function, we evaluated changes in several oxidative stress biomarkers in the RPE of rats after a short period of streptozotocin‐induced diabetes.

## 2 Materials and Methods

### 2.1 Chemicals and Antibodies

Streptozotocin was purchased from Enzo Life Sciences (ALX‐380‐010‐G‐001), antibodies were acquired from different distributors: ZO‐1 (Zymed‐Invitrogen, 61–7300), anti‐nuclear factor erythroid 2‐related factor 2, Nrf2 (Abcam, ab137550), mouse anti‐*β* actin (Abcam, ab8224), secondary antibodies Cy3‐conjugated anti‐rabbit (Chemicon, AP‐182C), horseradish peroxidase‐linked anti‐rabbit (NA934), and HRP anti‐mouse (NA931 Amersham). Chemiluminescent HRP substrate ECL (Millipore Corp, Billerica, MA) was used. Electrophoresis chemicals and DC Protein Assay kit were purchased from Bio‐Rad Laboratories; glutathione Kit (Abcam ab239709); quercetin, L‐NAME (Nω‐nitro‐L‐arginine methyl ester hydrochloride N‐5751), nitroblue tetrazolium chloride (NBT), bovine serum albumin (A‐7888 for immunohistochemistry and A‐9647 for Western Blot), and all other chemicals were sourced from Sigma‐Aldrich‐Merck. Glycated hemoglobin (HbA1c) was determined using a DCA Vantage analyzer (Siemens).

### 2.2 Experimental Animals

The breeding stock of Long–Evans rats was purchased from Charles River Laboratories, USA. Female adult Long–Evans rats (170–200 g) were housed under standard laboratory conditions (21°C± 1°C, 12 h light–dark cycle starting at 7:00 a.m.) and had free access to food and water.

### 2.3 Induction of Diabetes

Diabetes was induced by a single intraperitoneal injection of streptozotocin (STZ) (98 mg/kg) dissolved in a 0.1 mM citrate–phosphate buffer (pH 4.5) [18].

Normal (non‐diabetic) rats received vehicle alone. Some of the normal and diabetic rats were treated with either quercetin or L‐NAME to evaluate their possible protective effects against oxidative/nitrosative stress. Quercetin (QE, Sigma Chemical, St. Louis, MO, USA), dissolved in dimethyl sulfoxide—0.9% saline, was administered intraperitoneally (10 mg/kg) 5 days a week starting 1 day after STZ injection.

Control rats were injected with the same volume of dimethyl sulfoxide—0.9% saline. Similarly, Nω‐Nitro‐L‐arginine methyl ester (L‐NAME, Sigma Chemical), dissolved in saline, was administered intraperitoneally (50 mg/kg) 5 days a week, starting 1 day after the STZ injection. Control rats received the same volume of 0.9% saline. Insulin was not administered to any experimental group.

Animals were divided randomly into 12 groups:

Group 1:

Vehicle control animals (30).

Group 2:

Vehicle control animals with L‐NAME (3).

Group 3:

Vehicle control animals with quercetin (3).

Group 4:

STZ 7 days (11).

Group 5:

STZ 7 days with L‐NAME (3).

Group 6:

STZ 7 days with quercetin (3).

Group 7:

STZ 20 days (28).

Group 8:

STZ 20 days with L‐NAME (3).

Group 9:

STZ 20 days with quercetin (3).

Group 10:

STZ 45 days (27).

Group 11:

STZ 45 days with L‐NAME (3).

Group 12:

STZ 45 days with quercetin (3).

Blood glucose levels were measured using a blood glucose monitor (Accu‐Check Active, Roche). Animals were considered diabetic and included in this study if blood glucose exceeded 250 mg/dL (90% of injected animals reached this parameter); the few remaining were excluded. This group does not allow blinding selection, as all the animals with or without treatment were used.

All injected animals in each group were sacrificed, along with age‐matched normal rats, after 7, 20, and 45 days after STZ administration. They were decapitated without anesthesia, as it has been reported to increase oxidative stress; also, because the samples are used for other experiments, including glycine receptors, which interact with anesthetics. The eyes were removed and hemisected, the anterior segment and the retina were discarded, and the retinal pigment epithelium–choroid complex (RPE) was carefully peeled away using fine forceps. All efforts were made to minimize animal suffering, pain, and distress and to reduce the number of rats used. We tried to optimize tissue samples to be used in this and other experiments.

We state that the study was conducted in accordance with the Mexican Institutes of Health Research rules (DOF. NOM‐062‐Z00‐1999) and the National Institutes of Health Guide for the Care and Use of Laboratory Animals (NIH Publication No. 80–23, revised 1996), and was approved by our Institutional Animal Care and Use Committee (CICUAL, Institute of Cellular Physiology of the Universidad Nacional Autónoma de México) under Protocol Number RSS18‐14. We also state that all experimental procedures were performed according to the Animal Research: Reporting of in vivo Experiments (ARRIVE) guidelines.

### 2.4 Immunohistochemistry

The enucleated eyes were fixed in 4% paraformaldehyde in 0.1 M phosphate—0.9% NaCl, pH 7.4 (PBS). After 30 min, the eyes were hemisected, and the anterior part, including the retina, was eliminated. The eye cups containing the retinal pigment epithelium–choroid complex (RPE) were fixed for another 30 min. After rinsing in PBS, samples were permeabilized in 1% bovine serum albumin (BSA), 0.1% Triton X‐100 in PBS. Then, they were incubated overnight at 4°C with a rabbit antibody against ZO‐1 (1:200, Santa Cruz Biotechnology). This was followed by a 2 h incubation with a secondary Cy3‐conjugated anti‐rabbit (1:500, Zymed). In controls, a primary antibody was omitted. RPE flat mounts were mounted on glass slides and visualized using a Nikon microscope (Nikon Corp., Tokyo, Japan) and photographed with a Nikon DXM1200 digital camera (Nikon Corp., Tokyo, Japan).

### 2.5 Lipid Peroxidation

Lipid peroxidation was estimated by the levels of conjugated dienes (CD), as described previously [19, 20]. CD concentration was determined by its absorbance at 233 nm, using an extinction coefficient of 2.52 × 10–4 M‐1.

### 2.6 Ascorbic Acid Content

Ascorbic acid was determined by a spectrophotometric method based on the reduction of 2,6‐dichlorophenolindophenol, according to Omaye et al. 1979 [21].

### 2.7 Superoxide Dismutase Assay

Activity of the superoxide dismutase (SOD) was measured as described by Kono [20, 22]. The method utilizes tetrazolium salt to quantify superoxide radicals produced by hydroxylamine. One unit of enzymatic activity refers to the amount of enzyme required to inhibit the NBT reduction by 50%.

### 2.8 Glutathione Assay

Total and reduced glutathione (GSH) levels were determined by using a commercial kit from Abcam (ab239709). The assay is based on a kinetic enzymatic recycling method that detects the oxidation of GSH by 5, 5′ dithiobis‐2‐nitrobenzoic acid (DTNB) and glutathione reductase to measure total GSH [23]. Absorbance was measured at 412 nm in a microplate reader.

### 2.9 Na^+^/K^+^‐ATPase Activity

RPE were homogenized with Tris buffer, and the Na^+^/K^+^‐ATPase activity was assayed as described before [24, 25]. The enzyme activity was established by the production of inorganic phosphate from ATP in the presence and absence of ouabain.

### 2.10 Western Blot and Carbonylated Proteins

Protein expression levels were assessed by Western blot assay as formerly published [20]. Tissues were homogenized in lysis buffer containing protease and phosphatase inhibitor cocktail. Equal amounts of samples were resolved on a 10% SDS polyacrylamide gel, and the protein bands were transferred to polyvinylidene difluoride membranes (Millipore, USA). After blocking with 5% fat‐free milk, the membranes were probed with specific antibody (anti‐nuclear factor erythroid 2‐related factor 2, Nrf2; dilution, 1:800; Abcam, ab137550) at 4°C overnight. Then, membranes were incubated with horseradish peroxidase‐linked secondary antibody (dilution 1:10,000) for 60 min at room temperature. The protein bands were detected by enhanced chemiluminescence using chemiluminescent HRP Substrate (Millipore Corp, Billerica, MA). Blots were then stripped and re‐probed with monoclonal primary antibody anti‐β actin, Abcam ab 8224 (dilution 1:2000) to examine equal loading amounts. Levels of protein expression were quantified by densitometric analysis of specific bands on an Alpha DigiDoc RT (Alpha Innotech, San Leandro, CA) and analyzed using a densitometry program (AlphaEase FC StandAlone; Alpha Innotech, San Leandro, CA).

Levels of carbonylated proteins were assessed by Western blot analyses using a commercial kit (OxyBlot Protein Oxidation Detection Kit, Millipore), as previously reported [20]. Carbonyl‐hydrazine derivatized proteins were separated by electrophoresis, followed by Western blotting using a primary antibody specific to hydrazone and a secondary antibody that can be detected using a chemiluminescent HRP substrate. Densitometry was carried out as described above.

### 2.11 Protein Content Determination

Total protein content was determined with a commercial assay kit (BioRad DC) using BSA as the standard. Glucose concentration in blood was determined using a blood glucometer (glucose monitor Accu‐check, Roche). Glycated hemoglobin (HbA1c) was determined using a DCA Vantage analyzer (Siemens).

### 2.12 Statistical Analysis

All data are expressed as the mean ± SEM; the significance of the difference among the groups was assessed by one‐way ANOVA followed by Tukey's analysis. All statistical analyses were performed using the GraphPad Prism 9 software (La Jolla, CA, USA). A significant difference between the control and each experimental group was defined as *p ≤ 0.05.

## 3 Results

Diabetic phenotype was confirmed in accordance with our previous reports by impaired growth, polyuria, elevated blood glucose [15], and elevated HbA1c (Table 1). High glucose blood levels and the content of glycated hemoglobin induced by STZ were not modified by QE or L‐NAME treatment (Table 1).

**TABLE 1:** Blood glucose and HbA1c levels in the normal and diabetic rats.

Treatments	Glucose	HbA1c (%)
Control	136 ± 10	3.72 ± 0.12
STZ 20 days	459 ± 38*	6.37 ± 0.64*
STZ 45 days	453 ± 40*	10.70 ± 0.58*
STZ + QE 20 days	454 ± 42*	nd
STZ + QE 45 days	452 ± 54*	12.80 ± 1.0*
STZ + L‐NAME 20 days	470 ± 36*	nd
STZ + L‐NAME 45 days	466 ± 35*	11.5 0 ± 1.0*

*Note:* Blood glucose levels are expressed in mg/dl. STZ, streptozotocin; QE, quercetin; L‐NAME, Nω‐nitro‐L‐arginine methyl ester. Values are the average ± SEM from three to nine rats per group. *p ≤ 0.5 with respect to control. Nd, not determined.

**FIGURE 1:**
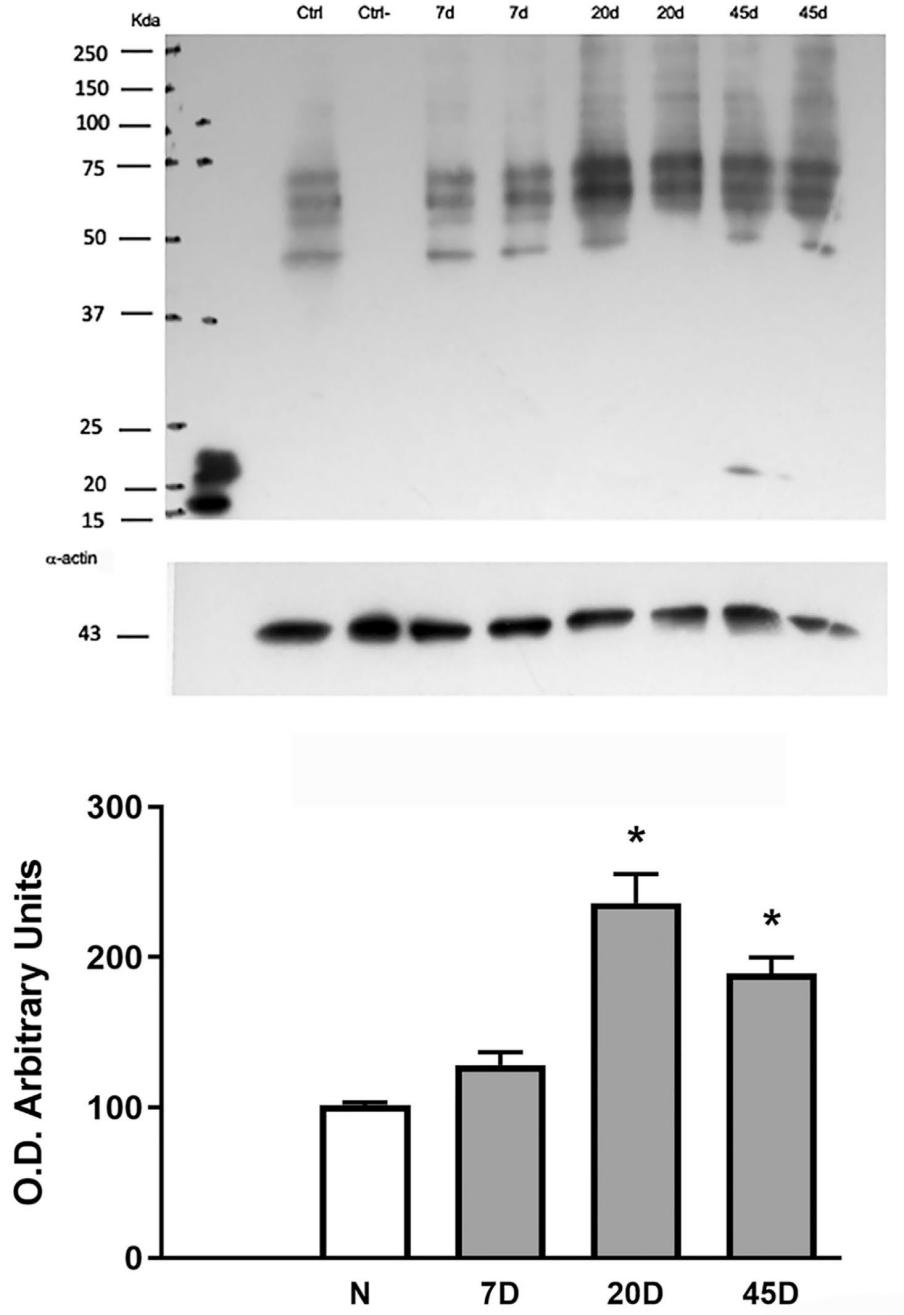
Carbonylated proteins in RPE. Upper part, a representative Western blot using the Oxyblot protein oxidation kit. Lower part, graphic of relative values of oxidized proteins from N (normal rats, *n* = 6), 7D (rats after 7 days of streptozotocin administration, *n* = 8), 20D (20 days, *n* = 12), and 45D (45 days, *n* = 11). Values are the mean ± SEM, *p ≤ 0.5 with respect to the normal.

First, we evaluated the levels of oxidized proteins, which are key targets of oxidative stress. Immunoblotting experiments showed a relatively high level of carbonylated proteins in the RPE from normal rats, which were considerably increased (twice) at 20 and 45 days after STZ administration (Figure 1). Unexpectedly, these levels were not modified by Quercetin or L‐NAME treatment (Figure S1).

Production of radical oxygen species (ROS) induced by hyperglycemia was determined by lipid peroxidation, which was estimated by the levels of conjugated dienes (CD). Levels of CD were relatively high in the RPE from normal rats (30 ± 13 nmoles/mg protein), which were around threefold higher than those in the retina [17]. Levels of CD were increased by 60% in the RPE from 45‐day diabetic rats (Table 2).

**TABLE 2:** Superoxide dismutase, Na^+^/K^+^‐ATPase activity, and conjugated dienes in the RPE from normal and diabetic rats.

	SOD	CD	Na^+^/K^+^‐ATPase
Normal	100 ± 16	100 ± 16	3.0 ± 0.2
7 days diabetic	62 ± 7	nd	3.1 ± 0.7
20 days diabetic	81 ± 12	136 ± 15	2.9 ± 0.2
45 days diabetic	198 ± 9*	166 ± 9*	2.3 ± 0.3

*Note:* Values are expressed as the percentage relative to their normal value. Superoxide dismutase (SOD) 2.64 ± 0.56 U/mg protein. One unit of SOD activity refers to the amount of enzyme required to inhibit the NBT reduction by 50%. Normal conjugated dienes (CD) 30 ± 13 nmoles/mg protein. Na^+^/K^+^‐ATPase activity is expressed as μg Pi/mg protein. Each value is the mean ± SEM of 5–6 rats per group. *p 0.05 with respect to the normal. nd, not determined.

The SOD activity in normal RPE reached values of 2.64 ± 0.56 U/mg protein. SOD activity showed a twofold increase in the RPE from 45‐day diabetic rats, while normal values were observed at earlier days of STZ administration (Table 2).

We also examined the content of the cellular antioxidant ascorbic acid in RPE from normal and diabetic rats. Ascorbic content in the RPE from normal rats reached values of 13.3 ± 1.6 μg/mg protein (*n* = 3). These values did not change after 20 days of STZ treatment but were remarkably reduced (50%) after 45 days of induced hyperglycemia.

**FIGURE 2:**
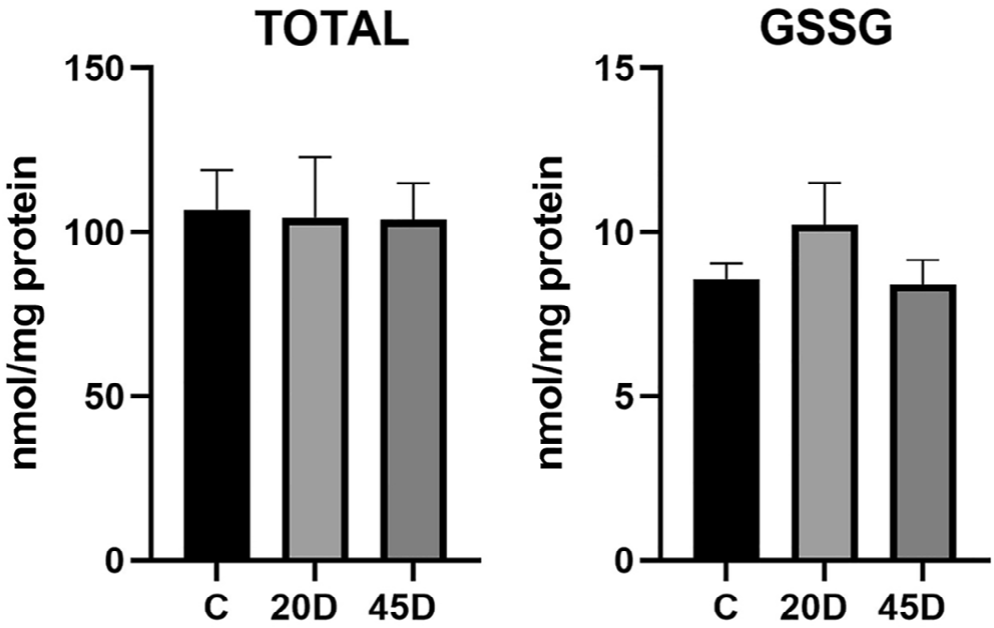
Glutathione levels in RPE from normal and diabetic rats. C (normal rats, *n* = 7), 20D (rats after 20 days of streptozotocin administration20, *n* = 5), and 45D (45 days, *n* = 6). The values were not significantly modified by hyperglycemic conditions, and are the mean ± SEM, *n* = 4–6 rats.

Then we analyzed the levels of GSH, which plays a main role in the antioxidant defense system in the cells. We found total GSH values of 179 ± 40 nmoles/mg protein in the RPE from normal rats, which were not statistically different from those of the diabetic rats (Figure 2). RPE levels of oxidized glutathione (GSSG) were very low (around 5%) in all conditions studied.

**FIGURE 3:**
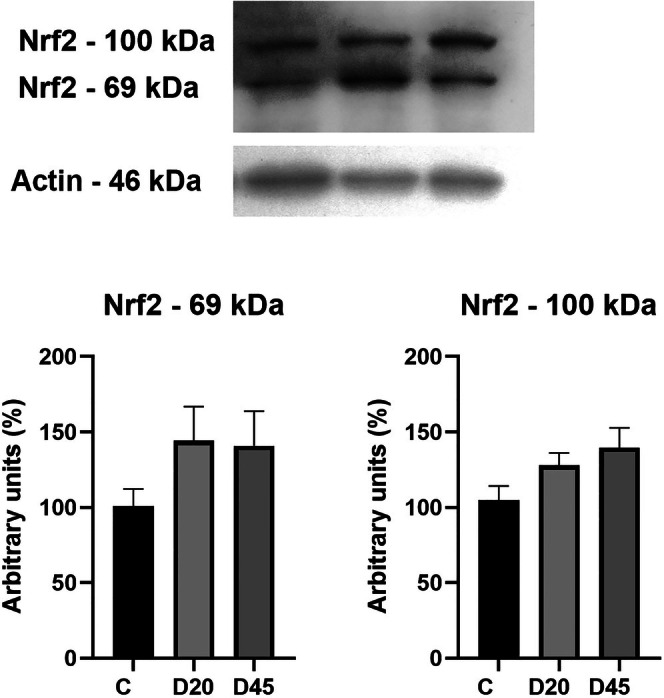
Nrf2 expression in RPE. Nrf2 Western blot in RPE from normal and diabetic rats. Immunolabeling revealed two bands, 68–70 kDa corresponding to its predicted molecular weight (55–65 kDa), and a second band of apparent molecular weight at 100 kDa. C (normal rats, *n* = 10), 20D (rats after 20 days of streptozotocin administration, *n* = 8), and 45D (45 days, *n* = 7). The values were not significantly modified by hyperglycemic conditions, and are the mean ± SEM, *n* = 6 rats per group.

Afterwards, we analyzed the expression levels of the nuclear transcription factor Nrf2, which plays a central role in cell response against oxidative damage. Nrf2 immunolabeling revealed two bands, one of ≈68 kDa corresponding to its predicted molecular weight (55–65 kDa), and a second band of apparent molecular weight of 100 kDa, which has been proposed as biologically relevant [26]. Interestingly, although there was an apparent increase in both 68–70 kDa and 100 kDa after 20 and 45 days of diabetes, it was not statistically significant (Figure 3).

In addition, we assessed RPE cellular structural integrity by means of ZO‐1 immunohistochemistry and Na^+^/K^+^‐ATPase activity. In normal RPE, Na^+^/ K^+^‐ATPase activity was found to produce 3.0 ± 0.2 μg Pi/mg protein. Na^+^/K^+^‐ATPase activity in RPE was not modified in the diabetic rats throughout the periods studied (Table 2).

**FIGURE 4:**
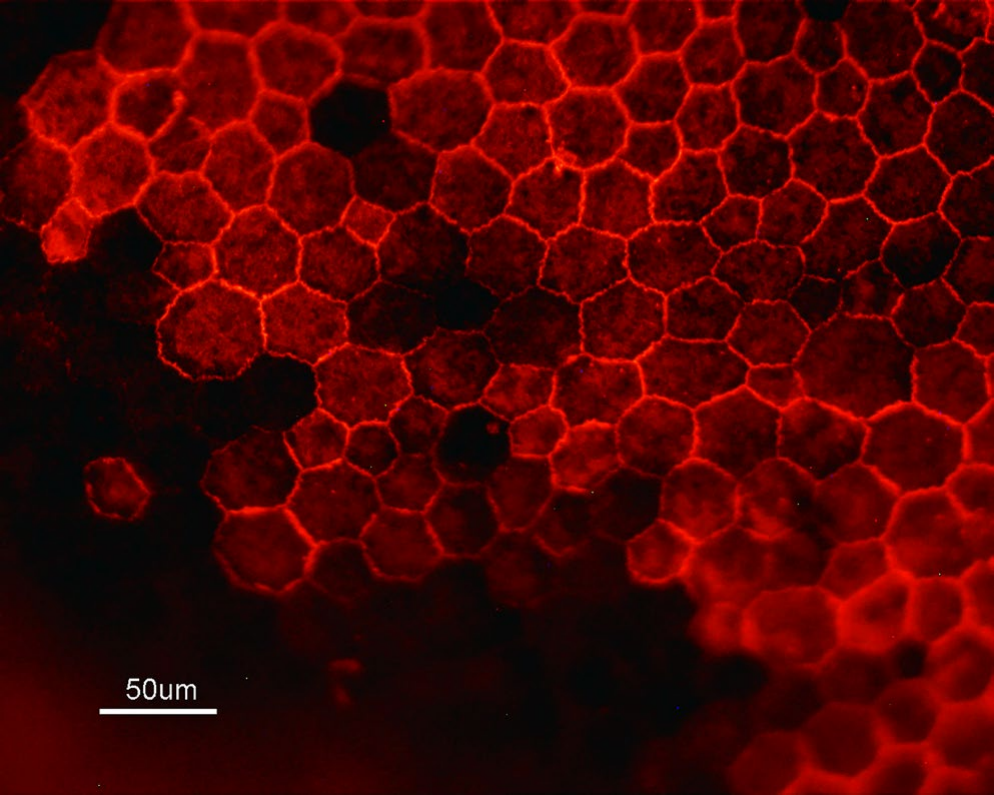
Immunohistochemical identification of ZO‐1 in isolated rat RPE after 20 days of diabetes induction. Loss of cells and discontinued cell–cell contacts are seen.

On the other hand, ZO‐1 staining indicated a disarray of hexagonal cells as well as loss of cells at 20 days after diabetes induction (Figure 4), indicating disrupted cell–cell interactions.

## 4 Discussion

DR is the leading cause of blindness in the world, affecting type 1 and type 2 diabetic patients, characterized by the breakdown of the BRB [1, 2, 3], although neuronal alterations have also been reported [4, 5, 6, 7]. The breakdown of the inner BRB is the focus of most research on the pathophysiology of DR. By contrast, the effect of diabetes mellitus on the RPE has received less attention. RPE plays an important role in retinal function by regulating the flow of metabolites and ions between the choroidal blood supply and the outer retina, as well as supporting photoreceptors [11, 12, 13]. Changes in RPE morphology, permeability, and electrophysiology in experimentally diabetic animals have been described, but little is known about metabolic alterations. Although there is evidence of oxidative stress and apoptosis in RPE cells incubated under high glucose concentrations [27, 28, 29], its demonstration in in vivo conditions has not been determined. Therefore, we analyzed the time course changes of several used biomarkers for oxidative stress in the rat RPE at early diabetes induction.

Protein oxidation is used as a marker for oxidative stress, and this oxidation leads to alterations in protein function and then to detrimental cellular activity. Relatively high levels of carbonylated proteins were observed in normal RPE, which were significantly increased since early hyperglycemia. Surprisingly, carbonylation levels were not modified by the administration of either quercetin or L‐NAME. Figure S1. Quercetin effects are contradictory, as it showed protective effects against oxidative stress in 2–6 months STZ diabetic rat retinas [30], and has potential therapeutic effects as an antioxidant in diabetic neuropathy and retinopathy [30, 31], as well as being effective in suppressing the photooxidation of the major component of lipofuscin A2E in RPE cells [32]. However, Quercetin did not modify levels of oxidized proteins in the retina at early diabetes induction [17]; moreover, this flavonoid was found to have deleterious effects on cell viability and induced cellular necrosis in human RPE primary culture cells [33].

On the other hand, the rise in carbonylated proteins can also be a result of the increase in NO and production of nitrated proteins [34]. In this respect, we previously found twofold higher NO content in RPE from diabetic rats compared to that in the normal rats [17], which might lead to protein carbonylation. In this line, the systemic nitrosative stress marker is associated with increased severity of DR [35]. Unexpectedly, we did not find a reduction of carbonylated proteins in the RPE from diabetic rats treated with the NOS inhibitor L‐NAME. These results might be explained by the L‐NAME doses we used.

We also analyzed lipid peroxidation levels in normal and diabetic rat RPE; we found relatively high levels of CD in the RPE from normal rats, which might be related to its photoreceptor outer segments' phagocytosis activity, since phospholipids in photoreceptors contain a high proportion of saturated fatty acids [36]. A significant increase in CD was only observed after 45 days of diabetes, which might suggest a high capacity of RPE to maintain its redox state and/or a fast lipid turnover. Interestingly, lipid peroxidation was accompanied by a reduction of ascorbate content. Ascorbate plays a central role in the antioxidative defense system by converting superoxide anions and lipid hydroperoxides into stable forms, thereby preventing lipid peroxidation [37, 38]. Ascorbate concentration has been reported to be high in the RPE and retina of several species [39, 40]. The presence of the specific vitamin C transporter SVCT in RPE and retina has been reported [41], which is impaired by high glucose concentrations [42, 43]. Moreover, a significant reduction in ascorbate uptake in RPE was observed after STZ‐induced diabetes [41]. Our results suggest that high glucose levels compete for ascorbic acid uptake, leading to a decrease in the supply of the antioxidant, which, at the times we studied, is not sufficient to produce oxidative stress because of the high RPE capacity to maintain its redox homeostasis. However, under continuous hyperglycemic conditions and/or low oxidative fluctuations, lipids and protein turnover may be affected, which can impact its function, leading to oxidative stress and further RPE damage.

Furthermore, the activity of the antioxidant enzyme SOD showed a considerable increase at 45 days of hyperglycemic condition, which most likely represents a response to maintain the cellular redox state. Indeed, SOD activity has been demonstrated to play a protective role in retinal capillary cell death and in DR [44].

Besides, levels of reduced GSH, a major contributor to cellular antioxidant capacity, were not significantly modified by hyperglycemic conditions, suggesting that the cellular redox state is maintained. Our results indicate that the RPE possesses a high capacity to generate reducing equivalents. Indeed, increasing glucose from 5.6 to 30 mM caused a six‐ to sevenfold increase in CO_2_ production by the Pentose Phosphate Pathway (PPP) in diabetic RPE [45]. The PPP generates NADPH, which can prevent oxidative stress by reducing glutathione via glutathione reductase.

In agreement with these results, the expression levels of both Nrf2 detected bands (68 and 100 kDa) were not modified under the hyperglycemic conditions we studied, suggesting that RPE possesses a high capacity to maintain its redox state.

Furthermore, we did not find changes in the activity of Na, K‐ATPase, which maintains an electrochemical gradient, crucial for various cellular processes, including cell volume and retinal adhesion. These results are contrary to those that found a loss of Na^+^/K^+^‐ATPase activity within the RPE from alloxan‐diabetic rabbits [46], and with a decrease in the amplitude of the c‐wave component of the electroretinogram generated by the RPE, observed in pigmented Long‐Evans rats at the 2‐week of diabetes [47]. However, the immunohistochemistry studies revealed alteration in RPE morphology, disrupted cell–cell interactions, and loss of cells at early diabetes induction, suggesting alterations in the outer BRB, which can lead to retinal injury. Although oxidative stress disassembles the tight junction of RPE cells [48], we found a high capacity of RPE to avoid oxidative stress.

The increase in oxidized proteins can also be a result of its accumulation, indicative of alterations in the cell degradation mechanisms and/or a reticulum unfolded protein response (UPR). In this respect, endoplasmic reticulum stress has been reported to occur in retinal endothelial cells and retina from diabetic mice [49, 50, 51]. Mechanisms that deserve additional studies.

Taken together, our results suggest that RPE possesses a high capacity to maintain its redox homeostasis; however, other mechanisms, such as UPR and/or protein turnover, might be disturbed under hyperglycemia, affecting RPE function and further damage, which in turn might lead to retinal function impairment.

## Author Contributions

Morales‐Ramírez E performed most of the Oxyblot experiments and analyzed part of the data; Villeda‐González JD performed some of the Oxyblot, Nrf2, and glutathione experiments and analyzed part of the data; Sánchez‐Chávez G performed some of the Oxyblot and HbA1c levels experiments, analyzed the data, and made the figures; and Salceda R conceived the project, analyzed the data, and wrote the manuscript.

## Ethics Statement

We state that this study was conducted in accordance with the National Institutes of Health Guide for the Care and Use of Laboratory Animals (NIH Publication No. 80‐23, revised 1996), and it was approved by our Institutional Animal Care and Use Committee (CICUAL, Institute of Cellular Physiology of the Universidad Nacional Autónoma de México) under Protocol Number RSS18‐14 in accordance with the Mexican Institutes of Health Research rules (DOF. NOM‐062‐Z00‐1999). We also state that all animals were treated in adherence to the ARRIVE 2.0 guidelines from Animal Research: Reporting of In Vivo Experiments (https://arriveguidelines.org, published in PLOS Biology in July 2020).

## Conflicts of Interest

The authors declare no conflicts of interest.

## Supporting information


**Figure S1a:** fba270067‐sup‐0001‐FigureS1a.pdf.

**Figure S1b:** fba270067‐sup‐0002‐FigureS1b.pdf.

## Data Availability

Stored in repository.
