# Supplementary figures and images for: Diabetes Induces Accumulation of Carbonylated Proteins in the Rat Retinal Pigment Epithelium Independently of Oxidative Stress
Source: FASEB Bioadv. 2026 Jan 21;8(1):e70067. doi: 10.1096/fba.2025-00230 (PMC12824452; doi:10.1096/fba.2025-00230)

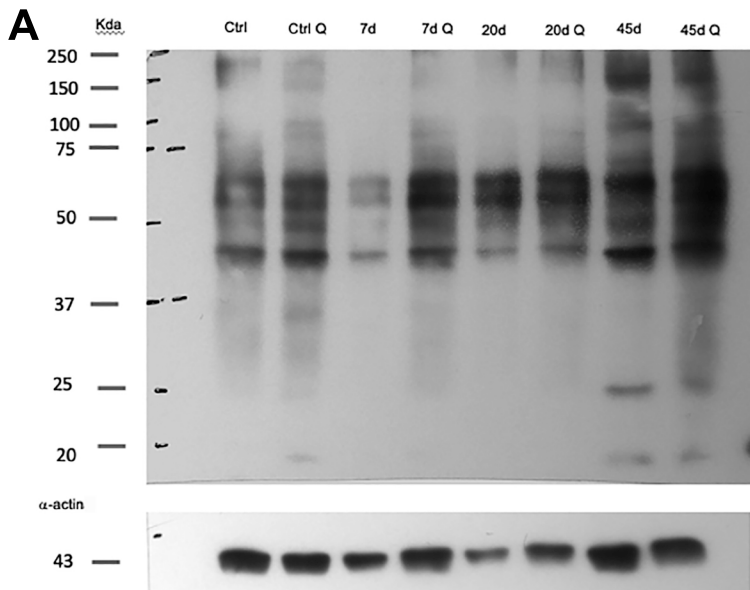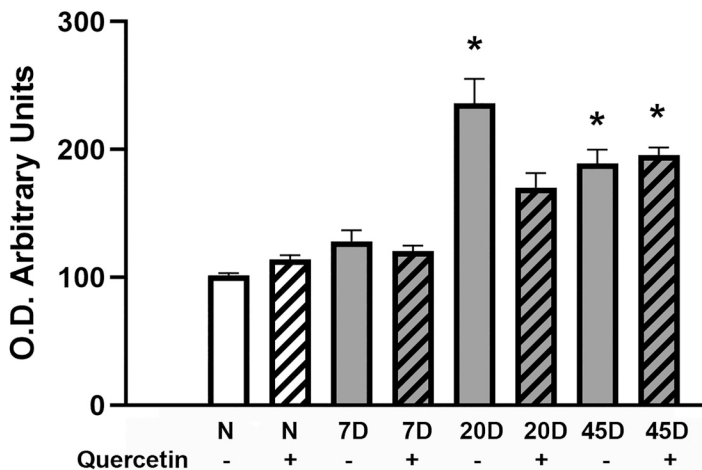

Supplement: Supplementary file 1 — Figure S1a: fba270067‐sup‐0001‐FigureS1a.pdf. [file FBA2-8-e70067-s002.pdf]

**B**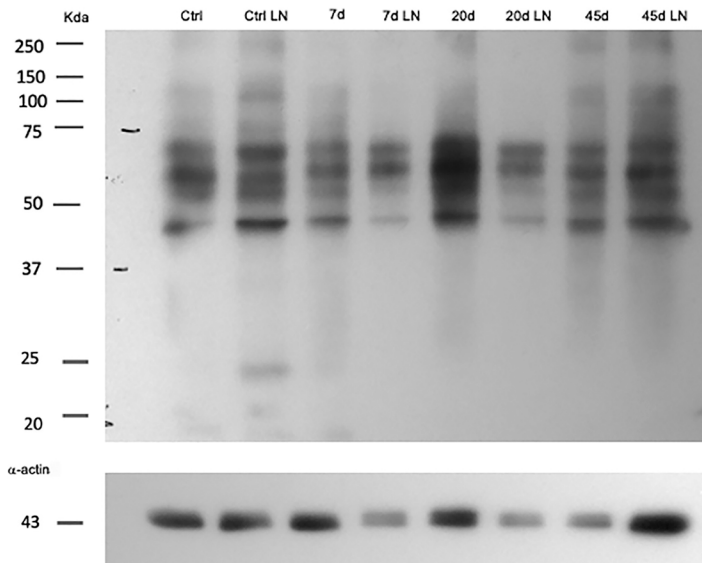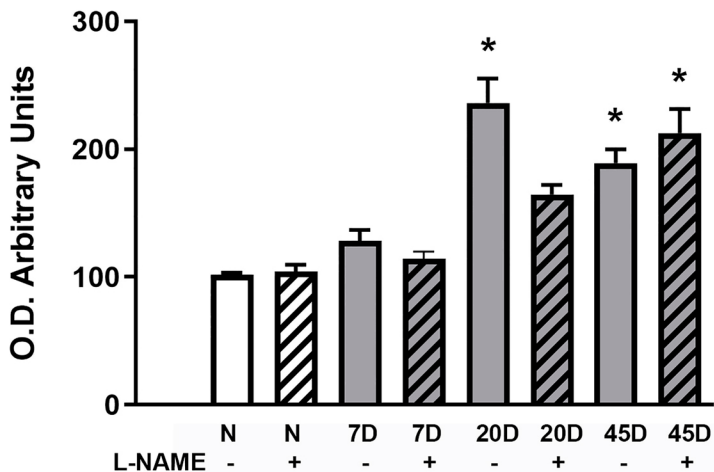

Supplement: Supplementary file 2 — Figure S1b: fba270067‐sup‐0002‐FigureS1b.pdf. [file FBA2-8-e70067-s001.pdf]
